# New Zealand supereruption provides time marker for the Last Glacial Maximum in Antarctica

**DOI:** 10.1038/s41598-017-11758-0

**Published:** 2017-09-25

**Authors:** Nelia W. Dunbar, Nels A. Iverson, Alexa R. Van Eaton, Michael Sigl, Brent V. Alloway, Andrei V. Kurbatov, Larry G. Mastin, Joseph R. McConnell, Colin J. N. Wilson

**Affiliations:** 1 NMBG/EES Department, New Mexico Tech, Socorro, NM USA; 2 EES Department, New Mexico Tech, Socorro, NM USA; 3 U.S. Geological Survey, Cascades Volcano Observatory, Vancouver, WA USA; 4 Division of Hydrological Sciences, Desert Research Institute, Reno, NV USA; 5 Paul Scherrer Institute, Villigen, Switzerland; 6 School of Environment, The University of Auckland, Private Bag, 92019 Auckland, New Zealand; 7 Centre for Archaeological Science (CAS), School of Earth and Environmental Sciences, University of Wollongong, Wollongong, NSW 2522 Australia; 8 Climate Change Institute, University of Maine, Orono, ME USA; 9 SGEES, Victoria University, PO Box 600, Wellington, 6140 New Zealand

## Abstract

Multiple, independent time markers are essential to correlate sediment and ice cores from the terrestrial, marine and glacial realms. These records constrain global paleoclimate reconstructions and inform future climate change scenarios. In the Northern Hemisphere, sub-visible layers of volcanic ash (cryptotephra) are valuable time markers due to their widespread dispersal and unique geochemical fingerprints. However, cryptotephra are not as widely identified in the Southern Hemisphere, leaving a gap in the climate record, particularly during the Last Glacial Maximum (LGM). Here we report the first identification of New Zealand volcanic ash in Antarctic ice. The Oruanui supereruption from Taupo volcano (25,580  ±  258 cal. a BP) provides a key time marker for the LGM in the New Zealand sector of the SW Pacific. This finding provides a high-precision chronological link to mid-latitude terrestrial and marine sites, and sheds light on the long-distance transport of tephra in the Southern Hemisphere. As occurred after identification of the Alaskan White River Ash in northern Europe, recognition of ash from the Oruanui eruption in Antarctica dramatically increases the reach and value of tephrochronology, providing links among climate records in widely different geographic areas and depositional environments.

## Introduction

**Figure 1:**
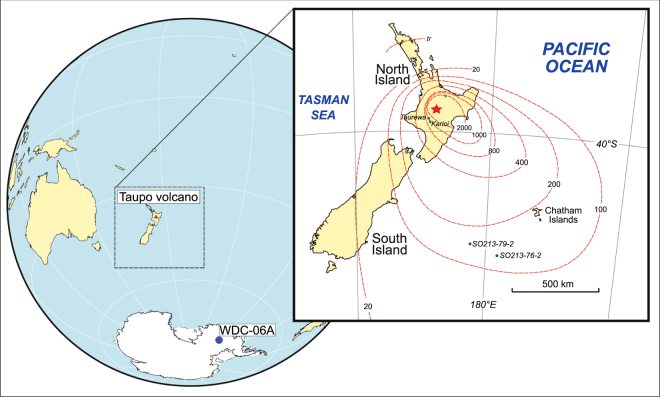
Location map showing the position of the WDC06A ice core (79°28.058′S, 112°05.189′W), along the location of the Taupo Volcano (red star, superimposed on Lake Taupo). Inset map shows locations of terrestrial or marine (green dots) and ice core samples (blue dot), as well as isopachs, in mm, for tephra from the Oruanui eruption, where 0 represents trace deposition. Map (after Alloway *et al*.^10,43^) created using Adobe Illustrator CS5.

Antarctic ice sheets capture and archive detailed climate records reaching as far back as ~1.5 Ma^1^. These records are recovered through deep ice core drilling and layer-by-layer analysis of the ice. In this study we focus on a high-resolution core (WDC06A), recovered from central West Antarctica (Fig. 1), which reached a final depth of 3405 m in December 2011. The ice fabric in this core suggests that its stratigraphic layering has remained undisturbed, providing a robust and complete climate record^2^. The WDC06A ice core has already provided the basis for important insights into changes in the carbon cycle during postglacial warming, as well as phasing of abrupt climate change events between the northern and southern polar regions^3–5^. Sulfur dioxide emitted during volcanic eruptions, and introduced into the stratosphere, converts to sulfate aerosol, which can produce short-lived global cooling^6^. One of the exciting new datasets provided by the WDC06A ice core is the sulfur-based record of volcanism, supplemented by electrical conductivity and particle counter measurements, which, when integrated with a Greenland ice record, provide insight into volcanic forcing of climate fluctuations^7^. In a related paper, Sigl and others^8^ integrated the excellent sulfur record provided by the WDC06A ice core with those from other Antarctic ice cores to more accurately estimate volcanic sulfate loading during the Common Era. This framework, along with the very accurate (<1% error) WDC06A chronological information, generated by a combination of layer counting and compared with the Hulu Cave record^9^, provides the opportunity to search for silicate ash, or tephra, from major volcanic eruptions that have previously been missed in the Antarctic record.

Tephra from the largest class of volcanic eruption, termed “super-eruptions”, characterized by magma volumes of >450 km^3^of magma, or >1000 km^3^ of pyroclastic deposit^10^, present good detection targets in ice cores, and the Oruanui supereruption from Taupo volcano (Fig. 1), New Zealand, is the prime target because of its notable size and occurrence during the last glacial period. The Oruanui is the second largest eruption globally in the past 100 kyr, producing ~1,100 km^3^ of tephra over a period of weeks to months^11^. The deposits (known regionally as the Kawakawa-Oruanui, or KOT, tephra) are fine grained and exceptionally widely dispersed as a result of the highly explosive interaction between magma and water from a large lake^12^, and associated groundwater system. A detailed study of ^14^C ages gathered from optimal material producing 8 ages from syn-eruptive material^13^. We have recalculated these ages using SHCal13 calibration^14^ (using OxCal v.4.3.2), which yielded an eruption age of 25,580  ±  258 cal a BP (±2 S.D.). This age is statistically indistinguishable from the age of 25,360  ±  160 and 25,358  ±  162 cal a BP reported in previous works^13,15^, which represent integrated ages from above and below the tephra, and were calibrated using IntCal09/SHCal09. These ages place the Oruanui eruption at an important time in earth’s recent climate history – the early LGM. This time period is central to our understanding of leads and lags in the global climate system, especially between the northern and southern hemispheres. Despite the large magnitude of this event, and evidence for widespread dispersal, tephra from the Oruanui eruption has not been previously identified in any Antarctic or Arctic ice cores. Here, we report the identification of particles from this significant eruption in ice core WDC06A, and consider the implications for poleward transport of tephra from volcanic eruptions.

## Methods and Results

**Figure 2:**
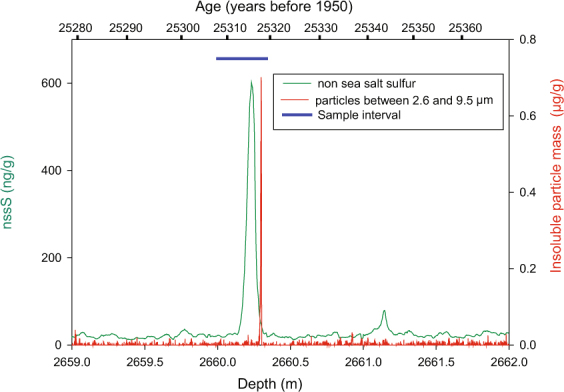
Non-sea-salt sulfur and particle concentrations (2.6 to 9.5 micron) measured in the WDC06A ice core. The nssS component was determined by removing the sea salt sulfur, based on the Na concentrations as a seawater tracer^7^. Cryptotephra was analyzed in an archived sample from 2660.000 to 2660.335 m.

Tephra layers in the WDC06A ice core were identified visually, or by using a downhole optical logger, and/or by observation of high particle counts during continuous melting analysis at the Desert Research Institute Trace Chemistry Laboratory (Reno, Nevada, USA) (method summarized in ref.^16^). A large sulfate spike and presence of particles 5–30 µm in diameter were recognized at 2660.3 m depth (Fig. 2), which corresponds to an age of 25,318 calendar years before 1950, with an estimated error of 1%^9^. This combination of significant particle and sulfur signals, and their relative timing in the ice record, pointed towards a large-magnitude eruption during the LGM, and the age of the layer is statistically indistinguishable from that of the Oruanui supereruption (25,580  ±  258 cal. a BP). Two ice samples from a depth range of 2660.000–2660.335 m were obtained from the National Ice Core Laboratory (Denver, Colorado, USA), melted, filtered and prepared for geochemical analysis following techniques of Dunbar and Kurbatov^17^. Particles were examined using scanning electron microscopy to document their size and shape; a routine procedure when investigating ice core tephra, in order to ascertain that volcanic material is present. Quantitative geochemical analysis was then carried out using the Cameca SX100 electron microprobe at New Mexico Tech^17,18^. As most of the particles were  <20 µm across, beam sizes of <20 µm were used for some particles, but beam sizes slightly larger than the glass particle was used. This process resulted in low analytical totals (mostly between 90 and 100%) due to overlap on epoxy, requiring analyses to be normalized to 100 wt.%. Studies suggest that this technique is effective at minimizing the unavoidable Na loss associated with analyzing small particles, and does not adversely affect the analytical accuracy, although the precision for a set of points may be slightly decreased^19^. Duplicate samples were prepared for microbeam geochemical analysis, and these were analyzed separately during different analytical sessions. A set of secondary standards, including rhyolitic and comenditic glasses VG568 and KN18 were analyzed during both analytical sessions to monitor accuracy and precision. In addition, four known samples of tephra from the Oruanui eruption sampled from well-documented terrestrial and marine sites were analyzed by electron microprobe using the same instrument and methods as the ice core samples. All data are presented in Supplementary Information S1.

**Figure 3:**
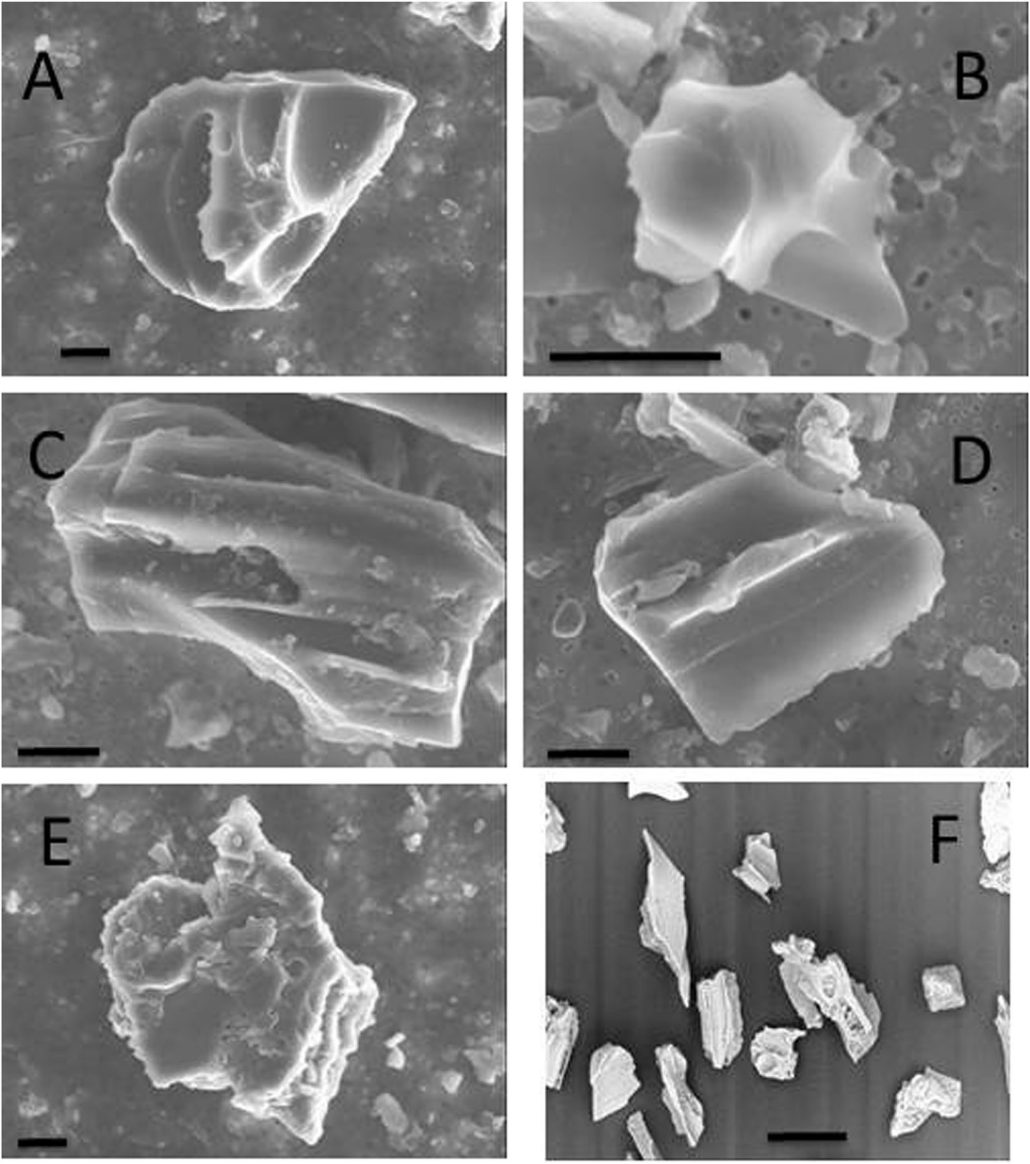
(**A–E**) Representative secondary electron microscope images of tephra filtered from sample 2660.000. The glass particles show equant to platy morphologies and round to elongated vesicles. Scale bars are 5 microns. (**F**) Secondary electron images of glass shards from near-source tephra deposits from the Oruanui eruption (sample location in decimal degrees 38.54055S, 176.07601E), sampled on land. Scale bar is 100 microns.

The two WDC06A ice core samples from 2660.3 m depth contained glass shards 5–30 µm long, with generally blocky morphology (Fig. 3A,B). Some contain vesicle walls (Fig. 3), or stretched vesicles (Fig. 3C,D), commonly with chipped or abraded margins. Others have an overall mossy to cuspate morphology (Fig. 3E), lacking groundmass crystals. These observations are indicative of the products of explosive volcanism and are broadly consistent with characteristics of terrestrial tephra from the Oruanui eruption (Fig. 3F), particularly with respect to the blocky morphology, elongate vesicles, and mossy shards^12,20^.

**Figure 4:**
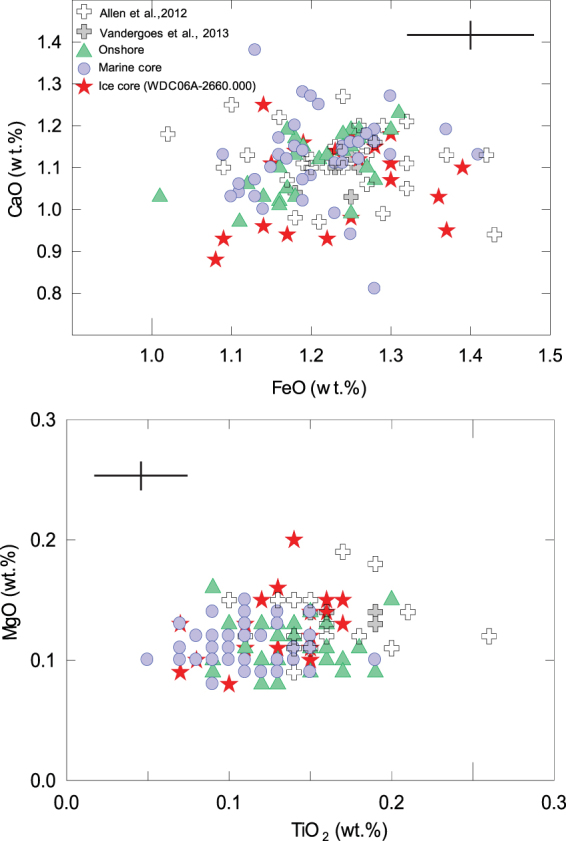
Individual glass shard compositions for tephra derived from  the Oruanui eruption, compared to the chemical composition of tephra analyzed from the WDC06A ice core. Cross represents analytical precision (see Supplementary Information S1). Data from Allan *et al*.^﻿21﻿^ and Vandergoes *et al*.^13^ are glass shard analyses from terrestrial samples of tephra from the Oruanui eruption, sampled at known localities. Location for “onshore” and “marine” samples (provided by author Alloway) are shown in Fig. 1.

Geochemical analyses reveal that glass shards from the WDC06A core samples are rhyolitic, chemically uniform, and, for key correlation elements such as Ca, Fe, and Ti, are indistinguishable in composition from the distal onshore and offshore tephra from the Oruanui eruption^13,21^ (Fig. 4, Supplementary Information S1 Table 1). Due to the small grain size of the ice core tephra, and need to carry out analyses with beam sizes smaller than 20 µm in diameter, Na loss took place during analysis, along with associated ingrowth of Si and Al, making these elements unsuitable for correlation purposes. The closest geochemical match is with material from the final (tenth) phase of the Oruanui eruption^11^ (Fig. 3), which generated the most voluminous and widespread fall deposits in the eruption. Another large New Zealand eruption, the Rotoiti, produced the Rotoehu tephra, which is geochemically^22^ similar to, but distinguishable from, the geochemical composition yielded by the WDC06A rhyolitic tephra. Furthermore, the Rotoiti eruption, with an age of 47.4 ± 1.5 ka^23^ does not represent a potential match to the WDC06A rhyolitic tephra, which has an age of 25,318  ±  250 calendar years before 1950, whereas the age of the Oruanui eruption (25,580  ±  260 cal a B.P.) is statistically indistinguishable. Most known Antarctic tephra from this age range are trachytic^17,24^, and no rhyolitic Antarctic tephra of an appropriate age are known. The striking similarity of age and geochemistry and indistinguishable age between tephra produced by the Oruanui eruption and that found in the WDC06A ice core at 2660.3 m depth strongly support this correlation.

**Figure 5:**
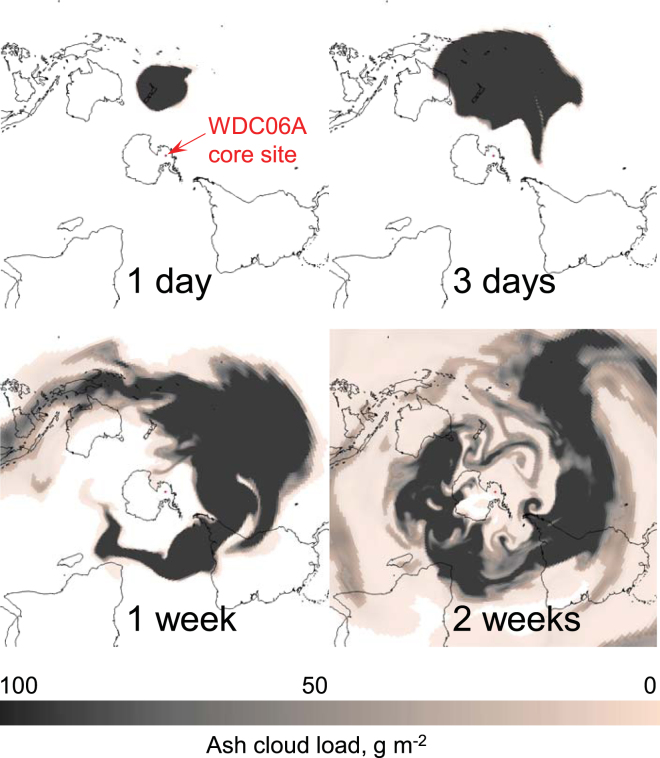
Ash3d simulation showing transport of volcanic ash from an Oruanui-sized eruption of Taupo volcano, New Zealand. Color scale gives ash load in the atmosphere (g m^−2^) at 1 day, 3 days, 1 week, and 2 weeks after the start of eruption. Note location of the WDC06A ice core in West Antarctica. Initially, the umbrella cloud expands radially, covering all of New Zealand and is ultimately swept into the polar jet, enveloping the Southern Hemisphere and depositing trace amounts of ash over Antarctica. Maps created using the atmospheric transport model Ash3d^25^.

To test the plausibility of tephra transport from New Zealand to West Antarctica, we simulated a large-magnitude eruption using the atmospheric transport model Ash3d^25,26^. Inputs are based on unit 10 of the Oruanui eruption from Taupo volcano, as established from field-based studies of the eruption deposits^11,12^. We assumed an eruption rate of 1.5  ×  10^9^ kg s^−1^, similar to the 1991 eruption of Mount Pinatubo, Philippines, and duration of three days, resulting in a total erupted volume of 150 km^3^ (dense-rock equivalent). The ash transport was modeled as an expanding umbrella cloud with a maximum height of 20 km above sea level and a lower bound of 15 km. These characteristics are consistent with large-eddy simulations of water - influenced, coignimbrite-style volcanism from previous work^27^. To account for the physics of a gravity-driven intrusion, Ash3d adds a radial wind field within the umbrella cloud, producing an outward expansion rate proportional to the eruption rate^26^. We used two particle classes. The larger size class comprises 95% of the erupted mass (diameter 500 µm, density 1.8 g/cm^3^), representing coarser particles and ash aggregates that fall out close to the volcano. The remaining 5% represents the fine particles (diameter 20 µm, density 2.2 g/cm^3^) that escaped aggregation and underwent long-distance transport^28^, with characteristics similar to cryptotephra recovered from the ice core. The 3-D time-varying atmospheric structure (including temperature and horizontal and vertical winds) was initialized from the 2.5 degree NCEP/NCAR Reanalysis2 dataset (https://rda.ucar.edu/datasets/ds091.0/). We identified 80 time periods within this dataset that fit within the plausible range of LGM conditions during the time of the Oruanui eruption, with northwesterly flow over New Zealand (Kidson type ‘Blocking HE’) and strong circumpolar westerlies around Antarctica^29–32^ (see Supplementary Information S2). The model grid covered the entire globe from 88^o^S to 88^o^N with a nodal spacing of 1 degree in the horizontal and 2 km in the vertical (see Supplementary Information S2 Table 1). Results show that 100% of the simulations transport volcanic ash to the WDC06A ice core site in West Antarctica within days to weeks of eruption (S2 Table 2). The findings suggest that an eruption of this magnitude from Taupo would have readily deposited 20 µm tephra particles over the West Antarctic ice sheet, regardless of variations in the wind field and season (Fig. 5, and animation in Supplementary Information S3).

## Discussion

Overall, volcanic ash from non-Antarctic eruptions has been challenging to locate and geochemically fingerprint in Antarctic ice^17,33–36^. A number of Antarctic volcanoes have produced explosive eruptions over the past several 100,000 years, and these dominate the ice-hosted tephra record. Tephra from Antarctic volcanoes is typically alkaline in composition, and the tephra record in West Antarctica is dominated by trachytic-composition tephra from two West Antarctic stratovolcanoes, Mt. Berlin, and Mt. Takahe^17,24^. Few explosive rhyolitic eruptions have occurred in Antarctica^37^, and none have been recognized in ice cores from either West or East Antarctica^17,33^. In some well-dated parts of ice cores, tephra from large, non-Antarctic eruptions are suspected to be present based on the visual identification of shards, but the grain size (mostly  <  3 µm) is less than that of background terrestrial dust, and too small for geochemical analysis^36^. Our identification of cryptotephra from the Oruanui eruption, with some particles up to 20–30 µm in diameter, shows that a non-Antarctic eruption is capable of delivering large particles to the Antarctic ice sheet. Model results demonstrate that it may only take a few days to weeks for such large particles to be transported thousands of kilometers in strong winds aloft, which is consistent with the European record of Icelandic cryptotephra^38^. Simulations of ash transport confirm that the particles could have feasibly been transported from New Zealand, and our geochemical and morphological comparisons indicate that the particles are sourced from the Oruanui eruption.

In this case, the question arises—what factors influenced tephra transport to Antarctica, >5,000 km from source, in detectable amounts? We suspect that a number of factors were at play. First, the overall dispersal axis for the Oruanui deposit is to the southeast of New Zealand (Fig. 1)^13^, suggesting that the background winds were favorable for poleward transport at the time of eruption. Numerical modeling shows how the tephra would be caught up in the polar jet, and continue encroaching inland to the West Antarctic ice sheet over a period of days to weeks (Fig. 5). Similarly, it has been shown that tropospheric winds could transport continental dust from New Zealand to West Antarctica in as little as 3–5 days^39^. Second, and most importantly, the large magnitude of the eruption would have overwhelmed the background atmosphere. Of global eruptions in the past 100 kyr, the Oruanui is second only to the 74 ka Youngest Toba Tuff in terms of erupted volume^11^. During high-intensity eruptions, the ash column rises vertically through the atmosphere and spreads outward as an umbrella cloud. These clouds can expand radially for hundreds of km regardless of the background winds, as observed during the 1991 eruption of Pinatubo (Philippines)^26,40^. Our modeling indicates that an Oruanui-sized eruption would produce an umbrella cloud thousands of km in diameter, transporting tephra and gas radially in all directions (Fig. 5). This gravity-driven transport would significantly enhance the overall transport distance of large particles, maintaining them aloft over much greater distances than would be possible by passive transport in the ambient wind field.

Recognition of a cryptotephra layer from the Oruanui supereruption as an LGM time-marker in a deep Antarctic ice core opens the door to direct correlations between phasing and climate shift precursors contained in terrestrial, marine and glacial environmental records. We identify this layer, comprising particles up to 30 µm diameter, as geochemically linked to the most voluminous, final phase of the Oruanui eruption from New Zealand, suggesting that the eruption’s dispersal power and interaction with the background wind field enabled transport to the Antarctic >5,000 km away. Such travel distances for eruptive clouds depositing cryptotephra are not unusual, and have demonstrably been exceeded (up to 7000 km or more along main wind dispersal directions) for moderate^41^ and large^42^ explosive eruptions.

Although this cryptotephra appears to be exceptional within the WDC06A ice core, in terms of its larger grain size and possibly higher particle concentration, its presence demonstrates that other non-Antarctic tephra layers may be present and recognizable. This particular layer will serve as a valuable early LGM time-stratigraphic marker in the emerging Antarctic tephrochronological framework. Further refined particle recognition and sample preparation techniques may allow more of these well-dated tephra layers to be identified in Antarctic ice, allowing further direct correlations between different, intercontinental, sedimentary records, and providing golden spikes for the Antarctic ice chronology.

## Electronic supplementary material

Supplementary Information S1

Supplementary Information S2

Supplementary Video
