# Supplementary material for: New Zealand supereruption provides time marker for the Last Glacial Maximum in Antarctica
Source: Sci Rep. 2017 Sep 25;7:12238. doi: 10.1038/s41598-017-11758-0 (PMC5613013; doi:10.1038/s41598-017-11758-0)
Supplement: Supplementary file 2 — Supplementary Information S2 [file 41598_2017_11758_MOESM2_ESM.doc]

Supplementary Information (S2) for **New Zealand supereruption provides time marker for the Last Glacial Maximum in Antarctica**, by Nelia W. Dunbar^1^, Nels A. Iverson^2^, Alexa R. Van Eaton^3^, Michael Sigl ^4,5^, Brent V. Alloway^6, 7^, Andrei V. Kurbatov^8^, Larry G. Mastin^3^, Joseph R. McConnell^4^, Colin J.N. Wilson^9^

Supplementary discussion

Supplementary Table 1

Supplementary Table 2

Caption for Supplementary Animation

**Supplementary discussion**

Input parameters for ash transport modeling

One goal of this study is to test the plausibility of ash transport from New Zealand to Antarctica during the Oruanui eruption of Taupo volcano. The eruption source parameters (e.g., erupted volume, ash plume structure) are reasonably well constrained from field studies (Wilson 2001; Van Eaton and Wilson 2013) and large-eddy simulations of eruption behavior (Van Eaton et al. 2012). These characteristics are reflected in the inputs shown in Supplementary Tables S1-S2. In contrast, the atmospheric structure during the Oruanui eruption has never been directly addressed. The eruption occurred ~25.4 thousand years ago, during the early Last Glacial Maximum (LGM). In general, proxy data from New Zealand and elsewhere in the Southern Hemisphere indicate colder, drier and winder conditions during this time period relative to modern-day climate. But more specifically, the overall dispersal direction of the Oruanui ash deposits provides clues about prevailing weather patterns during the time of eruption. Ash was dispersed primarily to the southeast (Fig. 5), indicating northwesterly flow over New Zealand. Based on the Kidson (2000) framework of synoptic weather analysis, these conditions are most likely to occur in association with Blocking type weather pattern HE (Lorrey et al., 2012; their Fig. 9). The existence of a blocking pattern is overall consistent with proxy data presented by Lorrey and Bostock (2017) suggesting that the Oruanui eruption occurred within an interstadial – a brief period of relatively mild climate, nested within a colder LGM.

To constrain the wind fields used in our simulations, we have taken the simple approach of ‘smart sampling’ a modern-day weather dataset for the paleo-conditions described above. We interrogated the NCEP Reanalysis2 dataset (<http://dss.ucar.edu/pub/reanalysis2>) for 72-hr time slices between 1948 and 2016 during which Kidson type Blocking HE prevailed over New Zealand according to the dataset of Renwick (2011). Eighty time periods were identified and used to initialize the starting conditions in our ash transport modeling (Supplementary Table 2).

Supplementary Table 1. Input parameters for all Ash3d simulations of tephra transport and deposition used in this study, using Ash3d core code version 837. (*) Assumes magma density of 2,500 kg m^-3^ and deposit density 1,000 kg m^-3^. (**) Particle shape factor from Wilson and Huang (1979).

| *Domain characteristics* |  |
| --- | --- |
| Location of Taupo vent | -38.8200°, 175.9600° |
| Location of WDC06A ice core | -79.4677°, -112.0865° |
| Grid extent (degrees lat) | -88 to 88 |
| Grid extent (degrees lon) | 360 |
| Nodal spacing in horizontal (degrees) | 1 |
| Nodal spacing in vertical (km) | 2 |
| Number of nodes in x, y, z | 360 x 176 x 12 |
| *Eruption characteristics* |  |
| Total erupted volume, km^3^ DRE | 150 |
| Total erupted volume, km^3^ bulk* | 375 |
| Total erupted mass, kg* | 3.75E+14 |
| Eruption duration, hrs | 72 |
| Mass eruption rate, kg s^-1^ | 1.45E+09 |
| Simulation duration, hrs | 672 |
| Top of umbrella cloud, km asl | 20 |
| Base of umbrella cloud, km asl | 15 |
| Mass percent of coarse particles | 95 |
| Coarse particle diameter, microns | 500 |
| Coarse particle density, kg m^-3^ | 1800 |
| Mass percent of fine particles | 5 |
| Fine particle diameter, microns | 20 |
| Fine particle density, kg m^-3^ | 2200 |
| Particle shape factor, F** | 0.44 |

**Supplementary Table 2**. Wind field inputs and results for the 80 simulations using Ash3d. Note that all other parameters were held constant, as shown in Table S1. The simulation start times shown here were taken from the NCEP/NCAR Reanalysis2 (2.5 degree) dataset based on synoptic analysis to be 100% Kidson type ‘Blocking HE’ for the 72 hr duration of the eruption, after Kidson (2000). See Supplementary discussion for details. Cloud arrival indicates when the ash cloud arrives over the WDC06A ice core location, in hours after the start of the simulation. The ashfall deposit at the core site is also shown in mass per unit area at the end of each model run. (*) Denotes the simulation shown in Figure 5 of the main text and in the supplementary animation.

| Run ID | Start time, YYYY-MM-DD HH:mm:ssZ | Cloud arrival, hrs after start | Ashfall deposit at WDC06A, kg m^-2^ |
| --- | --- | --- | --- |
| 1 | 1948-11-08 00:00:00Z | 175.6 | 0.020 |
| **2*** | **1949-02-04 12:00:00Z** | **184.7** | **0.030** |
| 3 | 1949-05-24 00:00:00Z | 185.6 | 0.020 |
| 4 | 1954-11-20 12:00:00Z | 205.6 | 0.040 |
| 5 | 1955-08-21 12:00:00Z | 197.3 | 0.050 |
| 6 | 1955-11-30 12:00:00Z | 369.8 | 0.000 |
| 7 | 1956-09-21 00:00:00Z | 208.5 | 0.070 |
| 8 | 1957-01-23 12:00:00Z | 165.3 | 0.060 |
| 9 | 1959-03-07 00:00:00Z | 286.5 | 0.010 |
| 10 | 1961-04-12 00:00:00Z | 183.2 | 0.020 |
| 11 | 1961-08-21 12:00:00Z | 234.0 | 0.030 |
| 12 | 1961-09-04 00:00:00Z | 242.1 | 0.010 |
| 13 | 1961-10-23 12:00:00Z | 303.6 | 0.030 |
| 14 | 1961-12-28 12:00:00Z | 119.0 | 0.020 |
| 15 | 1962-04-28 12:00:00Z | 137.6 | 0.050 |
| 16 | 1962-04-30 00:00:00Z | 256.0 | 0.060 |
| 17 | 1962-05-16 12:00:00Z | 174.9 | 0.050 |
| 18 | 1962-11-21 00:00:00Z | 129.4 | 0.050 |
| 19 | 1964-06-29 12:00:00Z | 208.0 | 0.050 |
| 20 | 1964-07-01 00:00:00Z | 170.9 | 0.080 |
| 21 | 1965-04-16 00:00:00Z | 189.3 | 0.010 |
| 22 | 1966-08-20 12:00:00Z | 192.9 | 0.060 |
| 23 | 1966-12-17 12:00:00Z | 153.0 | 0.050 |
| 24 | 1966-12-19 00:00:00Z | 168.6 | 0.080 |
| 25 | 1968-08-09 00:00:00Z | 286.1 | 0.020 |
| 26 | 1969-07-21 12:00:00Z | 150.4 | 0.030 |
| 27 | 1971-06-21 12:00:00Z | 246.5 | 0.060 |
| 28 | 1972-08-08 00:00:00Z | 129.2 | 0.050 |
| 29 | 1974-04-02 00:00:00Z | 321.9 | 0.020 |
| 30 | 1974-09-04 12:00:00Z | 297.6 | 0.010 |
| 31 | 1977-11-08 12:00:00Z | 163.4 | 0.030 |
| 32 | 1977-11-10 00:00:00Z | 126.1 | 0.040 |
| 33 | 1978-04-24 00:00:00Z | 282.0 | 0.020 |
| 34 | 1978-04-25 12:00:00Z | 260.6 | 0.020 |
| 35 | 1978-04-27 00:00:00Z | 206.9 | 0.020 |
| 36 | 1978-04-28 12:00:00Z | 170.8 | 0.010 |
| 37 | 1979-0723 12:00:00Z | 211.2 | 0.030 |
| 38 | 1982-05-31 12:00:00Z | 282.8 | 0.020 |
| 39 | 1983-04-05 00:00:00Z | 155.8 | 0.020 |
| 40 | 1983-06-09 12:00:00Z | 268.9 | 0.030 |
| 41 | 1984-09-09 00:00:00Z | 244.3 | 0.040 |
| 42 | 1985-06-14 00:00:00Z | 110.0 | 0.100 |
| 43 | 1985-06-15 12:00:00Z | 85.9 | 0.050 |
| 44 | 1985-06-17 00:00:00Z | 162.9 | 0.030 |
| 45 | 1985-08-07 00:00:00Z | 211.6 | 0.020 |
| 46 | 1986-10-13 12:00:00Z | 269.6 | 0.050 |
| 47 | 1989-07-31 00:00:00Z | 219.8 | 0.050 |
| 48 | 1989-08-01 12:00:00Z | 194.9 | 0.040 |
| 49 | 1989-08-03 00:00:00Z | 159.3 | 0.040 |
| 50 | 1990-10-17 12:00:00Z | 362.1 | 0.020 |
| 51 | 1990-10-19 00:00:00Z | 325.6 | 0.030 |
| 52 | 1991-03-30 12:00:00Z | 146.8 | 0.020 |
| 53 | 1991-08-28 12:00:00Z | 219.4 | 0.080 |
| 54 | 1991-08-30 00:00:00Z | 191.0 | 0.080 |
| 55 | 1994-07-31 00:00:00Z | 137.6 | 0.030 |
| 56 | 1995-11-29 00:00:00Z | 207.2 | 0.020 |
| 57 | 1995-12-10 00:00:00Z | 78.6 | 0.020 |
| 58 | 1966-04-25 12:00:00Z | 230.3 | 0.020 |
| 59 | 1966-06-04 12:00:00Z | 229.9 | 0.170 |
| 60 | 1998-07-06 12:00:00Z | 321.8 | 0.010 |
| 61 | 1998-07-08 00:00:00Z | 288.7 | 0.010 |
| 62 | 1998-10-06 00:00:00Z | 117.6 | 0.010 |
| 63 | 1999-07-31 12:00:00Z | 173.7 | 0.040 |
| 64 | 1999-11-05 12:00:00Z | 215.7 | 0.050 |
| 65 | 2000-10-27 00:00:00Z | 162.3 | 0.040 |
| 66 | 2001-11-30 00:00:00Z | 221.6 | 0.030 |
| 67 | 2003-07-25 00:00:00Z | 174.6 | 0.030 |
| 68 | 2003-08-05 12:00:00Z | 179.2 | 0.070 |
| 69 | 2003-08-24 12:00:00Z | 259.6 | 0.030 |
| 70 | 2003-10-08 00:00:00Z | 227.6 | 0.020 |
| 71 | 2004-09-09 00:00:00Z | 250.3 | 0.040 |
| 72 | 2005-07-27 12:00:00Z | 303.8 | 0.030 |
| 73 | 2006-04-15 12:00:00Z | 174.2 | 0.010 |
| 74 | 2006-04-20 00:00:00Z | 237.5 | 0.020 |
| 75 | 2009-04-24 12:00:00Z | 114.2 | 0.030 |
| 76 | 2009-06-08 00:00:00Z | 224.9 | 0.050 |
| 77 | 2009-06-09 12:00:00Z | 189.7 | 0.110 |
| 78 | 2009-08-21 12:00:00Z | 278.5 | 0.030 |
| 79 | 2010-06-17 12:00:00Z | 172.6 | 0.030 |
| 80 | 2011-04-21 00:00:00Z | 184.4 | 0.020 |

**Caption for Supplementary Animation**

Ash3d model results showing volcanic ash transport to Antarctica from Taupo Volcano, New Zealand during an Oruanui-sized eruption. The four-week simulation models a 72 hr-long eruption with a constant eruption rate of 1.5e9 kg s^-1^ and plume top of 20 km above sea level. Color scale gives airborne cloud load in metric tons per km^2^ (equivalent to grams per m^2^). Animation corresponds to Figure 5 in the main text (Run #2; see S2 Tables 1–2). Note how the ash cloud encircles Antarctica before encroaching inland, blanketing the entire Southern Hemisphere within days to weeks. Near the end of the simulation, the cloud dissipates as ash particles settle out of the atmosphere. Integrated Data Viewer (IDV) software from UCAR/Unidata was used in the analysis and visualization of the data [Unidata, 2012: Integrated Data Viewer (IDV) version 5.1u2 [software]. Boulder, CO: UCAR/Unidata. (http://doi.org/10.5065/D6RN35XM)].
